# Heterogeneity of Variances in Milk Yield in Murrah Buffaloes

**DOI:** 10.3390/ani15182686

**Published:** 2025-09-13

**Authors:** Raimundo Nonato Colares Camargo Júnior, Cláudio Vieira de Araújo, José Ribamar Felipe Marques, Marina de Nadai Bonin Gomes, Welligton Conceição da Silva, Tatiane Silva Belo, Carlos Eduardo Lima Sousa, Éder Bruno Rebelo da Silva, Larissa Coelho Marques, Mauro Marinho da Silva, Marcio Luiz Repolho Picanço, José de Brito Lourenço-Júnior, Alison Miranda Santos, Albiane Sousa de Oliveira, Jaqueline Rodrigues Ferreira Cara, André Guimaraes Maciel e Silva

**Affiliations:** 1 Postgraduate Program in Animal Science (PPGCAN), Institute of Veterinary Medicine, Federal University of Para (UFPA), Castanhal 68746-360, PA, Brazil; welligton.medvet@gmail.com (W.C.d.S.); eder.b.rebelo@gmail.com (É.B.R.d.S.); mauro.marinho@ifpa.edu.br (M.M.d.S.); joselourencojr@yahoo.com.br (J.d.B.L.-J.); alison@ufpa.br (A.M.S.); andregms@ufpa.br (A.G.M.e.S.); 2 Department of Agricultural and Environmental Sciences, Federal University of Mato Grosso (UFMT), Sinop 78550-728, MT, Brazil; cvaufmt@gmail.com; 3 Embrapa Eastern Amazon, Belém 66095-903, PA, Brazil; jrrffm@gmail.com; 4 Postgraduate Program in Animal Science, Faculty of Veterinary Medicine and Animal Science, Federal University of Mato Grosso do Sul, Campo Grande 79074-460, MS, Brazil; marina.bonin@ufms.br (M.d.N.B.G.); albianesousa45@gmail.com (A.S.d.O.); jaqueline.ferreira@ufms.br (J.R.F.C.); 5 Department of Veterinary Medicine, University Center of the Amazon (UNAMA), Santarém 68010-200, PA, Brazil; tatianebelovet@gmail.com (T.S.B.); cadu34.medvet@gmail.com (C.E.L.S.); 6 Department of Veterinary Medicine, University Center of the Amazon (UNAMA), Belém 66060-902, PA, Brazil; laracoel@gmail.com; 7 Federal Institute of Education, Science and Technology of Pará (IFPA), Santarém 68020-820, PA, Brazil; marcio.picanco@ifpa.edu.br

**Keywords:** milk production, genotype-environment interaction, genetic parameters, selection

## Abstract

The aim of this study was to assess the presence of heterogeneity of variance in milk yield in the first lactation of buffaloes and its subsequent influence on the genetic evaluation of Murrah breed sires. The analysis utilized a dataset comprising 2392 milk yield records of buffaloes involved in the Programa de Melhoramento de Búfalos do Brasil. The standard deviation classes were established by standardizing the averages of contemporary group levels, with positive values constituting the high standard deviation class and values equaling or less than zero comprising the low standard deviation class. The linear mixed model incorporated fixed effects of sire group, buffalo age at calving, and heterozygosity as covariates, along with additive genetic random effects. Variance components were estimated via Bayesian inference employing the Gibbs sampler to derive posterior means. The average posterior heritability obtained in analyses without considering heterogeneity of variances (i.e., the “general analysis”) was 0.21, while the averages 0.19 and 0.34 were obtained for the low and high standard deviation classes, respectively. The genetic correlation between standard deviation classes was 0.61. The genetic correlation estimates between the predictions of breeding values for milk yield were more closely aligned between the predictions obtained in the general analysis with the low standard deviation class, and more discrepant between the two standard deviation classes. In the animal genetic evaluation model, when heterogeneity of variance is disregarded, the variance components are substantially weighted towards the performance of individuals in the low phenotypic variability class. By disregarding the presence and heterogeneity of variance, the breeding values of the best sires were underestimated.

## Simple Summary

The aim of this study was to assess the presence of heterogeneity of variance in milk yield in the first lactation of buffaloes and its subsequent influence on the genetic evaluation of Murrah breed sires. The analysis utilized a dataset comprising 2392 milk yield records of buffaloes. The genetic correlation estimates between the predictions of breeding values for milk yield were more closely aligned between the predictions obtained in the general analysis with the low standard deviation class, and more discrepant between the two standard deviation classes. In the animal genetic evaluation model, when heterogeneity of variance is disregarded, the variance components are substantially weighted towards the performance of individuals in the low phenotypic variability class. By disregarding the presence and heterogeneity of variance, the breeding values of the best sires were underestimated.

## 1. Introduction

The strategic selection of animals can exert a direct influence on productivity and breeding systems, thereby serving as a pivotal instrument in the pursuit of optimized production efficiency [1,2]. The enhancement of the species’ desirable characteristics and the identification of individuals with superior genotypes are fundamental objectives of genetic selection [3]. The incorporation of selected animals into intensive production systems has been demonstrated to result in the generation of a substantial number of offspring, thereby ensuring the propagation of their desirable characteristics [4,5].

The most efficacious approach to enhancement is the utilization of selection based on the genetic evaluation of animals. This approach facilitates the identification of the most optimal individuals. However, this is not a rudimentary task, especially when it is necessary to accurately identify superior animals [6,7]. The most assertive diagnosis is also informed by the phenotype and genetic information, including data on parentage [8,9]. Genetic evaluation is a tool that has been demonstrated to guarantee the success of selection and to predict an individual’s breeding value [10,11].

Modern genetic evaluation employs more sophisticated statistical methods, such as the linear mixed model, which has the capacity to combine a variety of information, thereby facilitating a more extensive analysis of the breeding value of individuals. However, the primary limitation of this model is its inability to account for the effects of the interaction between genotype and environment [12,13]. In complex systems with a considerable impact from environmental variables, this failure to consider the interaction can adversely affect the productivity and performance of animals in numerous ways, as well as result in inaccurate classifications, which would affect the selection of individuals [14,15,16].

Genetic improvement programs must take the genotype–environment interaction into consideration. When animals with identical genotypes exhibit differential performance in disparate environments, the genetic classification may be inaccurate, potentially compromising the selection of individuals [17,18,19]. Consequently, evaluation models that incorporate this interaction are more pertinent, as they facilitate enhanced precision in the assessment of animal performance, taking into account environmental variability [20,21,22].

The genotype–environment interaction, or the manifestation of heterogeneity of variance, occurs when the same genotype varies according to environmental conditions, such as nutrition, management, or climatic conditions [21,23]. In production systems, this heterogeneity can result in different animal performances, making it difficult to select the best individuals for breeding [24,25]. However, the absence of consideration for these effects can lead to an underestimation or overestimation of genotypes, thereby compromising the efficacy of the breeding program [21,26].

In addressing this challenge, numerous programs have adopted models that explicitly consider this interaction as a means to circumvent the issue [27,28]. These models facilitate a more profound comprehension of the responses of genotypes in diverse environments [19,21]. Consequently, the selection of animals can be optimized to ensure superior performance, irrespective of their environmental context, thereby maximizing the genetic potential of individuals. The aim of this study was to assess the presence of heterogeneity of variance in milk yield and its repercussions on the genetic evaluation of Murrah breed sires.

## 2. Materials and Methods

Information was used from 2392 total milk yields in the first lactation records of buffaloes participating in the Brazilian Buffalo Improvement Program—PROMEBULL.

Total milk yield (TMP) was regressed on lactation length (LL). It was then corrected for 305 days (MP305) of lactation using the following expression: MP305 = TMPl + 5.47111 × (305 − LL).

Direct heterozygosity (HTZ) was estimated using breed information, taken as the value of the deviation from the Murrah breed. The HTZ was assumed to vary from zero (0) to one (1), as proposed by Diaz [20]. The HTZ value was calculated according to the equation proposed by Wolf et al. [19]:

HTZ = αtM αbO + αtO αbM

where

αtM αtO = Proportion of Murrah or other breed genes in the buffalo’s father;

αbO αbM = Proportion of Murrah or other breed genes in the buffalo’s mother.

The calving months were grouped into four seasons (CS) in order to adapt the analysis to regional climatic adversities, considered as follows: CS = 1 the period from January to March; CS = 2 the period from April to June; CS = 3 the period from July to September; and CS = 4 the period from October to December.

The groups of contemporaries were made up of a combination of the fixed effects of herd, year, and calving season. Contemporary group information with at least three observations was taken into account. The buffalo’s age at calving was used as a covariate (linear effect), with an average of 73.80 ± 36.61 months.

To study the heterogeneity of variance, classes of standard deviations (SD) were formed. To do this, we first obtained the MP305 averages for each contemporary group, which were then treated as a continuous variable and standardized with a mean of zero and a variance of one. Thus, positive values made up the high SD class and values equal to and less than zero made up the low SD class.

In this way, uni-characteristic analyses (general analysis), disregarding the heterogeneity of variances, and bi-characteristic analyses, in which milk yield in each standard deviation class was considered as a separate characteristic, were carried out.

The linear mixed animal model used is represented as follows: y = Xβ + Za + e, where “*y*” is a vector of observations, “*β*” is a vector of fixed effects (group of contemporaries, heterosis and buffalo age at calving), “*a*” is the vector of direct additive genetic effect and “*e*” is the vector of residual effect, “*X*” is the incidence matrix that associates “*β*” with “*y*”, and “*Z*” is the incidence matrix of the direct genetic effect.

The (co)variance components were obtained by Bayesian inference, using the Gibbs sampler through the GIBBS1F90 program [29]. To obtain posterior averages, chains of 600,000 cycles were used, saving every 20 cycles, with a discard period of 60,000 initial cycles. The Geweke criterion [30] was used to diagnose the chains at the 5% probability level.

To compare subsequent heritability means in each standard deviation class, the Z-test was applied at the 5% probability level, using the expression

$$Z_{i}=\frac{{\bar{l}}_{i}^{A}-{\bar{l}}_{i}^{B}}{\sqrt{\frac{S_{A}^{2}}{n_{A}}+\frac{S_{b}^{2}}{n_{B}}}},$$

where

${\bar{l}}_{i}^{A}$ and ${\bar{l}}_{i}^{B}$ are the posterior averages of heritabilities;

$S_{A}^{2}$ and $S_{b}^{2}$ are the variances associated with the posterior averages;

n_A_ and n_B_ are the sample sizes, in this case equal to 27,000 cycles.

Subsequent to acquiring the predicted breeding values for milk yield for the sires in the general analysis and in each SD class, the Spearman correlations were calculated between all the sires, as well as only for the sires with positive breeding values for milk yield in the general analysis.

The breeding values of the sires predicted for milk yield in the general analysis were also regressed in relation to the predictions obtained in each SD class. This was performed to observe possible underestimation or overestimation in the predictions of breeding values between genetic evaluation models that did or did not consider the presence of heterogeneity of variances.

The averages and standard deviations for milk yield in the general analysis and in each SD class, inclusive of the minimum and maximum values observed, along with the coefficient of variation for each weight, are presented in Table 1.

## 3. Results

The posterior means, standard errors, medians, credibility interval (CI), and Geweke’s diagnosis are presented for the additive and residual genetic variance components (Table 2). Additionally, the posterior means of heritability and genetic correlation for milk yield across the various phenotypic standard deviation classes are outlined in a general analysis.

Geweke’s diagnostic assessment indicated that the Markov chain sizes were appropriate for estimating the posterior means for all variance components (*p* > 0.05). The posterior mean of the additive genetic variance components exhibited an increase from the low to the high SD class, reflecting a similar trend to the posterior mean of the phenotypic variance.

The credibility interval of the posterior means of the additive and residual genetic variance between the classes and SD was evaluated, revealing that they do not intersect, thereby indicating the existence of heterogeneity of variance for milk yield in buffaloes.

Consequently, the posterior mean heritability values differed between the SD classes, with a greater increase in the posterior mean in the high SD class. Furthermore, the posterior mean obtained in the general analysis was close to that obtained in the low SD class.

The “Z” test was applied to compare the equality of posterior heritability means between the low and high SD classes. This analysis yielded an estimate of 2.35, with a probability of 0.019, indicating that the means differ from each other (*p* < 0.05). Therefore, the additive and residual genetic variances for milk yield differ from each other, and they provide different posterior heritability means, confirming the existence of variance heterogeneity.

The estimated additive genetic correlation (0.61 ± 0.001) for milk yield between SD classes indicates that they behave as distinct traits, confirming the presence of heterogeneity of variances (Table 3).

In the context of breeding value regression analysis, the sires’ predicted milk yield values were examined in relation to the predictions obtained within each standard deviation class. The regression equations obtained included y^ = −0.04474 + 1.14591X (R^2^ = 0.88) and y^ = 0.0085 + 0.66135X (R^2^ = 0.77) and were obtained for the low and high standard deviation classes, respectively.

**Figure 1:**
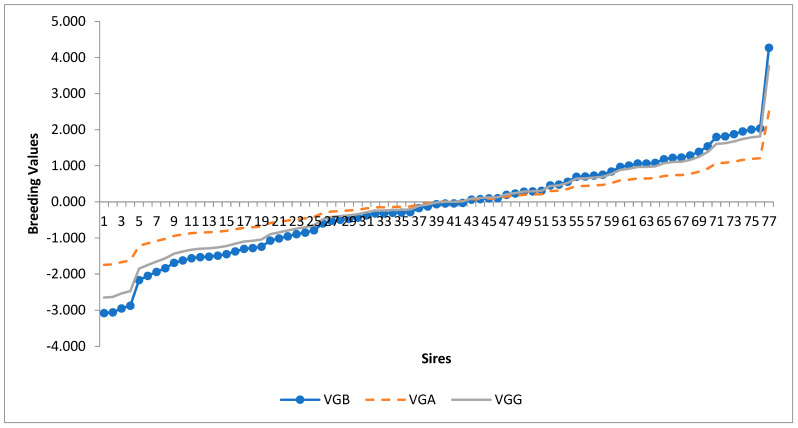
Breeding values of sires for milk yield regressed on general analysis (VGG) for the low SD (VGB) and high SD (VGA) standard deviation classes, with equations equal to y^ = −0.04474 + 1.14591X (R^2^ = 0.88), for the low standard deviation class.

Sires with positive breeding values for milk yield exhibit underestimation of predictions in the high SD class, while sires with negative predictions demonstrate the opposite behavior (Figure 1). The sire predictions obtained in the low SD class manifest a similar tendency to those observed in the general analysis.

Consequently, by neglecting the heterogeneity of variances in the genetic evaluation model for these animals, the variance components are disproportionately influenced by the performance of individuals in the low phenotypic variability class. This, in turn, results in predictions of breeding values that underestimate the potential of the best sires.

## 4. Discussion

The posterior mean of the genetic variance increased from low to high standard deviation (SD), which reflects a proportional increase in the variance of the phenotypes (Table 2). This suggests that as the phenotypic SD increases, the genetic variability is also more expressive [31,32]. This phenomenon is particularly evident in populations exhibiting high phenotypic variability, resulting in more pronounced genetic effects due to genetic–environment interactions and increased dispersion of the phenotypic value [33,34].

The credibility intervals of the posterior means of the additive and residual genetic variance do not overlap between the SD classes, which confirms the heterogeneity of the variances [35,36]. Milk yield in buffaloes exhibits distinct patterns of variation influenced by environmental factors. This heterogeneity can directly impact the accuracy of genetic evaluations, underscoring the necessity of employing models that account for this variability [37,38].

The disparity in average heritability between SD classes suggests a genetic influence on phenotypic expression (Shao et al., 2021; [39,40]). A higher heritability in the high SD class suggests that, under these conditions, selection based on breeding values may be more efficient. However, the proximity of the averages in general, and the low SD class, indicates the influence of phenotypic variability in masking relevant genetic differences [34,41].

The “Z” test confirmed a significant difference (*p* < 0.05) when comparing the heritability between the classes, thereby reinforcing the existence of heterogeneity in the variances and the influence on the genetic parameters [42,43,44]. This difference considers a more homogeneous model, which can lead to more erroneous estimates of the genetic variability of these individuals [45,46,47].

The additive genetic correlation of 0.61 + 0.001 between SD classes indicates that milk yield is a distinct trait with different levels of phenotypic variability [48,49,50]. This finding underscores the presence of variance heterogeneity and emphasizes the impact of this heterogeneity on sire selection, thereby affecting the classification of individuals based on their phenotypic variability [46,51,52].

When analyzing the Spearman correlation between the estimated breeding value in the different DS classes, it was observed that they are lower because they only consider breeders for milk production, suggesting that the classification may vary according to the heterogeneity of the variance, especially for those individuals that present better productive potential [53,54]. This is a salient factor, as it can result in the improper selection of sires if this heterogeneity of variance is duly considered [55,56,57].

The regression equations obtained for the breeding values found in this study demonstrated discrepancies in the SD classes. The equation for the low SD class exhibited a higher slope (1.14591) and a higher R^2^ (0.88), suggesting that the predicted breeding values in this class and in the overall analysis are more robust. On the other hand, the equation for the high SD class exhibited a lower slope (0.66135) and a lower R^2^ (0.77), indicating a weaker correlation between the breeding values estimated under these conditions.

Sires with positive breeding values for milk yield in general tend to be underestimated in the high SD class (Figure 1), while those with negative predictions show the opposite behavior [36]. These results are critical and imply that individuals with high genetic potential may be unfairly overlooked in the selection process if variance heterogeneity is not taken into account [46,58].

The failure to consider heterogeneity of variance in the genetic evaluation model can lead to an underestimation of the potential of sires and the ranking of individuals [59,60,61]. The extant results demonstrate the existence of phenotypic variability and its influence on the estimation of heritability, correlations, and genetic predispositions. This highlights the necessity for models that incorporate this factor, thereby ensuring more accurate evaluations in the selection of dairy sires in buffalo [51,62,63].

## 5. Conclusions

Heterogeneity of variance has been observed in the milk yield of Murrah buffaloes. In the animal genetic evaluation model that disregards heterogeneity of variance, the variance components are weighted much more heavily toward the performance of individuals in the low phenotypic variability class. Disregarding the presence and heterogeneity of variance results in an underestimation of the breeding values of the best sires. The present study corroborates the substantial presence and considerable ramifications of variance heterogeneity in milk production in Murrah buffaloes, particularly during the first lactation. In this sense, it is important to highlight the substantial disparity in the posterior heritability means between the different SD classes, with an average heritability of 0.19 being observed for the low SD class and a notably higher heritability (0.34) for the high SD class, in contrast to the mean obtained in the general analysis. This significant distinction clearly demonstrates that MP is influenced differently under different environmental conditions and levels of phenotypic variability. Furthermore, the non-overlapping of the credibility intervals of the additive and residual genetic variances between the SD classes serves to further reinforce the existence of this heterogeneity. It is imperative to acknowledge the presence and heterogeneity of variance when evaluating the genetic values of superior sires; failure to do so can lead to an underestimation of these values. The most critical consequence of ignoring this heterogeneity is that the variance components are disproportionately weighted in favor of the performance of individuals that fall into the class of low phenotypic variability. The existence of bias has been shown to result in a systematic underestimation of the genetic values of the most superior sires, particularly those that possess a high degree of productive potential and would excel in environments characterized by greater variability.

## Figures and Tables

**Table 1:** Number of contemporary group classes (NCG), number of records (N), estimated mean, estimated standard deviation (SD) and coefficient of variation (CV), in percentage, in the different phenotypic standard deviation classes.

Class of SD	NCG	N	Mean	SD	CV(%)	Confidence Interval (0.95)
Low	91	1562	2071.54	449.79	21.71	[1977.91; 2165.17]
High	70	830	2642.69	594.28	22.48	[2501.04; 2784.34]
General	161	2392	2269.72	573.13	25.25	[2180.56; 2358.88]

NCG for General (161) refers to total distinct CGs, whereas Low (91) and High (70) refer to the number of CGs whose standardized means were ≤0 or >0, respectively.

**Table 2:** Posterior means and standard errors, medians, credibility interval (CI), and Geweke’s diagnosis for the components of additive genetic variance (σ^2^a) and residual variance (σ^2^e), and posterior means of heritability (h^2^) and genetic correlation (rg) for milk yield in the different classes of phenotypic standard deviations in general analysis.

		Class of Standard Deviation		
	Parameters	Low	High	General
σ^2^a	Mean ± SD	353.51 ± 0.57	1134.22 ±1.59	472.47 ± 0.54
	Median	347.70	1119	467.60
	CI	178 a 543.30	637.30 a 1656	303.30 a 651.80
	Geweke	0.01	−0.01	0.01
σ^2^e	Mean ± SD	1480.62 ± 0.45	2180.15 ± 0.97	1804.00 ± 0.41
	Median	1478	2174	1803
	CI	1342 a 1633	1880 a 2503	1670 a 1940
	Geweke	0.001	0.01	−0.01
h^2^		0.19 ± 0.0004	0.34 ± 0.0003	0.21 ± 0.0002
r_g_		0.61 ± 0.001		

Note: Different letters (a) indicate statistical difference. CI is the 95% credible interval.

**Table 3:** Spearman correlation estimates between the breeding values for milk yield (above the diagonal) obtained in each SD class and in general analysis, among all 77 sires and for the 33 positive sires for milk yield in general analysis.

	N = 77 (100%)			
	Class of Standard Deviation			
		Low	High	General
N = 33 (43%)	Low	1	0.87	0.95
	High	0.78	1.00	0.73
	General	0.87	0.51	1.00

## Data Availability

The data presented in this study are available upon reasonable request from the corresponding author.
